# From Graphitized Coke Waste to Aminated Graphene Oxide: Enhancing Reactive Oxygen Functionalities Through Liquid-Phase Exfoliation Pretreatment

**DOI:** 10.3390/nano16211434

**Published:** 2026-11-06

**Authors:** Maiby Valle Orta, Edgar Cuara, Alfonso Mercado, Enrique Díaz Barriga Castro, Pablo González Morones, Marcos Granda, Diego Martínez, Patricia Álvarez, Uriel Sierra

**Affiliations:** 1 Laboratorio Nacional de Materiales Grafénicos, Centro de Investigación en Química Aplicada, Boulevard Enrique Reyna Hermosillo 140, San José de los Cerritos, Saltillo 25117, Coahuila, Mexico; maiby.valle@ciqa.edu.mx (M.V.O.); edgar.cuara@ciqa.edu.mx (E.C.); jesus.mercado@ciqa.edu.mx (A.M.); enrique.diazbarriga@ciqa.edu.mx (E.D.B.C.); pablo.gonzalez@ciqa.edu.mx (P.G.M.); 2 Instituto de Ciencia y Tecnología del Carbono INCAR-CSIC, C/Francisco Pintado FE, 26, 33011 Oviedo, Spain; mgranda@incar.csic.es; 3 Centro de Investigación en Geociencias Aplicadas, Nueva Rosita 26830, Coahuila, Mexico; diegomartinez@uadec.edu.mx

**Keywords:** coke, graphite, graphene, graphene oxide

## Abstract

The conversion of industrial carbon wastes into functionalized graphene materials represents an attractive strategy for the sustainable production of advanced carbon nanomaterials. In this work, a graphitized coke-like waste generated during metallurgical coke production was evaluated as a precursor for the preparation of graphene oxide (GO) and amine-functionalized graphene oxide, using a commercial synthetic graphite as a reference material. Prior to oxidation, both graphites were subjected to a mild aqueous liquid-phase exfoliation (LPE) treatment employing water as the sole processing medium. Graphene oxides were subsequently prepared using a modified Hummers method and functionalized with n-octylamine through an ultrasound-assisted reaction. X-ray diffraction confirmed the successful conversion of both graphitic precursors into graphene oxide through the appearance of the characteristic (001) reflection at approximately 10–11° (2θ). Structural characterization revealed that the LPE treatment promoted partial delamination of the graphitic crystallites while preserving graphitic crystallinity. FTIR and thermogravimetric analyses indicated that LPE modified the distribution and accessibility of oxygen-containing functionalities generated during oxidation. After amination, the LPE-derived materials exhibited more pronounced spectroscopic and thermal changes, suggesting a greater participation of reactive oxygen-containing groups during functionalization. The results demonstrate that graphitized coke waste can be transformed into graphene oxide and nitrogen-functionalized graphene materials with characteristics comparable to those obtained from commercial graphite, providing a simple and sustainable route for the valorization of industrial carbon residues into value-added graphene-based material.

## 1. Introduction

Graphene-based materials are among the most widely investigated two-dimensional (2d) systems, while other emerging 2d materials such as black phosphorus (bp) and Mxenes have further expanded the scope of this field [1,2]. Processing these bulk precursors into high-quality nanosheets relies primarily on top-down exfoliation technologies. Among this diverse landscape of 2D materials, graphene and its derivatives remain the primary platform for chemical functionalization.

Amino-functionalized graphenes have attracted widespread attention in advanced materials engineering due to the unique combination of basicity, high interfacial reactivity, and tunable surface energy that these moieties impart to the carbon framework [3,4,5,6]. In addition, amino groups provide anchoring sites for further chemical modification, enhance the compatibility of graphene with organic and polymeric matrices, and improve interfacial charge-transfer processes [5,6,7]. Their presence make the obtained materials key components in high-performance polymer nanocomposites [8,9], chemical and gas sensors [10,11], adsorbents for environmental remediation [12], and energy conversion or electrocatalytic devices [13,14,15]. To synthesize the amino-functionalized graphenes, two main routes are usually employed: (i) direct in-situ heteroatom doping (such as high-temperature chemical vapor deposition [16,17], solvothermal synthesis [13,18,19], or thermal annealing in ammonia atmospheres [4,20]) and (ii) post-synthetic covalent modification of pre-formed carbon structures [3]. Although direct doping methods effectively incorporate nitrogen atoms mainly into the graphitic lattice, they typically require harsh processing conditions, afford poor selectivity over the resulting chemical species, and cause extensive structural damage or loss of oxygen reactive sites at elevated temperatures [4,20,21]. In contrast, post-synthetic chemical modification using oxygenated intermediates—specifically the oxygen functional groups present in graphene oxide (GO)—provides a mild, highly selective, and versatile alternative [3,22]. In this approach, GO serves as a reactive intermediate whose oxygen-containing functional groups, distributed across the basal plane (epoxide -C-O-C- and hydroxyl -OH- groups) and edges (carboxylic acid -COOH- functionalities) of the sheet [23], provide diverse sites for covalent chemical functionalization. These oxygenated groups provide complementary routes for chemical functionalization, although their relative abundance and reactivity (and accessibility) depend strongly on the structure (i.e., the crystallinity of the graphite) and the preparation conditions of GO [22,23]. In particular, edge-carboxyl groups can be functionalized through amidation reactions, but their relatively low abundance limits the maximum achievable loading via this pathway [22]. By contrast, basal-plane epoxide groups are, in general, distributed over a much larger GO surface and are highly susceptible to nucleophilic attack, making epoxide ring opening an attractive route for covalent functionalization with amines and other nucleophiles [22]. Moreover, epoxide functionalization does not require the carboxyl activation steps commonly employed for amidation, although the reaction conditions may still require appropriate solvents, temperature, or catalysts [22]. It was recently demonstrated that primary amines react with epoxy functionalities in graphite oxide [24], with the reaction proceeding predominantly through the nucleophilic ring opening of epoxide groups rather than through amidation of carboxylic acids [7]. Based on those results one can conclude that basal-plane epoxide rings constitute key reactive sites for covalent grafting in this post-synthetic strategy. Primary alkylamines and related nitrogen nucleophiles can undergo SN_2_ nucleophilic ring opening of these epoxide moieties, yielding covalently bound beta-amino alcohol functionalities. Under optimized conditions, ultrasound-assisted tip sonication in alcoholic media has been reported to accelerate this reaction, enabling the formation of amino-alcohol-functionalized GO within short reaction times [25]. This has an effect in the ulterior utilization of these materials and the resulting alkylamine-functionalized GO exhibited enhanced compatibility with non-polar polymer matrices; for example, incorporation into bio-based polymyrcene rubber (PMy) has been reported to modify interfacial interactions and vulcanization behavior through the presence of the grafted alkylamine functionalities [26]. Furthermore, the alkylamine chain length can influence the interfacial compatibility and dispersion of the functionalized GO within the polymer matrix [27]. Therefore, the extent of GO amination is intrinsically linked to the density, accessibility, and spatial distribution of reactive epoxide functionalities within the GO precursor.

Conventionally, GO is synthesized via top-down chemical oxidation of graphitic precursors using the historical protocols of Brodie [28] or Hummers and Offeman [29]. Beyond conventional natural or commercial graphites, it was recently demonstrated that other parent materials as pre-graphitic carbon materials, industrial cokes or even coke-like wastes can also serve as effective, low-cost alternative precursors for producing high-quality graphene oxide derivatives [30,31]. Beyond the choice and stoichiometry of the oxidizing agents, it is well established that the intrinsic physical and crystalline characteristics of the starting carbon precursor—including parent graphite crystallinity [32] and particle or mesh size [33]—strongly condition both the oxidation mechanism and the resulting chemical structure. Furthermore, studies on physical pre-modifications, such as those by Sun and Fugetsu [34], showed that pre-expanding graphitic precursors creates a foam-like intermediate that facilitates oxidant penetration, resulting in a safer, faster, and more homogeneous conversion into graphene oxide.

Inspired by these fundamental insights, and considering that top-down liquid-phase exfoliation (LPE) has proven effective in disrupting interlayer van der Waals forces in diverse 2d crystals [1,2], we hypothesize that introducing a controlled mechanical pretreatment via liquid-phase exfoliation (LPE) prior to chemical oxidation can tailor the structural features of carbon precursors. Rather than attempting complete delamination into single-layer sheets, this gentle aqueous LPE pretreatment is designed to partially disrupt π–π layer stacking [35,36]. Moreover, it is intended herein to conduct the LPE using water as the only reagent and solvent, avoiding the use of toxic organic solvents such as N-methyl-2-pyrrolidone (NMP) or dimethylformamide (DMF), which are commonly employed in graphite exfoliation processes due to their favorable surface energy matching with graphene [36,37]. In recent years, increasing attention has been devoted to the development of environmentally benign LPE routes, including water-based exfoliation systems that enable the production of few-layer graphene while reducing the environmental and health concerns associated with conventional organic solvents [37,38]. With this pretreatment, it is expected to facilitate oxidant penetration to inner basal planes during subsequent Hummers oxidation, thereby promoting a more extensive chemical oxidation and maximizing the density of basal-plane epoxide groups (C–O–C) required for efficient amination. Another objective of this work is to evaluate this strategy on sustainable, low-cost carbon sources, such as coke-like industrial wastes, a high-temperature coking byproduct sourced from Nueva Rosita (Coahuila, Mexico), building upon the precursor concept established by Sierra et al. [30]. Therefore, we systematically investigate the effect of the pre-exfoliation treatment on graphitized coke-derived graphite (G-waste-derived), using a commercial graphite (SG) as a reference, on the structural organization and distribution of oxygen-containing functionalities generated during the subsequent Hummers oxidation. Particular attention is paid to the formation and accessibility of basal-plane oxygen groups, especially epoxide functionalities, which are expected to play a key role in the subsequent covalent amination reaction. By correlating precursor pre-exfoliation, oxygen functional group distribution, and amination efficiency, this work provides new insights into how mild aqueous liquid-phase exfoliation can tailor the chemical reactivity of graphene oxide. Furthermore, unlike previous studies focused mainly on the use of coke-derived materials as carbon precursors, this work demonstrates that graphitized coke can be successfully converted into graphene oxide and amino-functionalized graphene materials with characteristics comparable to those obtained from commercial synthetic graphite. Beyond the specific oxidation and functionalization strategy investigated here, the present results provide a basis for the future development of more direct graphene production routes from coke-derived feedstocks and contribute to the valorization of industrial carbon residues into value-added graphene-based materials.

## 2. Materials and Methods

### 2.1. Materials Used

A commercial graphite powder (<20 µm) was supplied by Sigma Aldrich (SG, Darmstadt, Germany). A High-temperature waste from a coke oven (CkH) obtained from Nueva Rosita, México. CkH contains 11.3% of ashes with composition Al (40–45 wt.%), Fe (39–42 wt.%), Ca (13–15 wt.%) and, at a lower extent, Mg (1–2 wt.%) and Zn (0.5–1.5 wt.%), as determined by ICP-Ms. KMnO_4_ was purchased by Fermont, hydrogen peroxide (H_2_O_2_) (30%) was bought from J. T. Baker (Radnor, PA, USA), hydrochloric acid (HCl), (n-octylamine) and other solvents were supplied by Sigma Aldrich (SG, Darmstadt, Germany).

### 2.2. Preparation of Graphene Materials

#### 2.2.1. Preparation of the Graphite from Mexican High Temperature Coke-like Waste

High-temperature coke-like waste (CkH) obtained from Nueva Rosita, México, was sieved through a 0.75 µm mesh. The resulting powder was graphitized at 2800 °C in a graphite furnace for 45 min under an argon flow to produce graphite (which was labeled as G-waste-derived).

#### 2.2.2. Pretreatment of Graphites via Liquid Phase Exfoliation

In this stage, 5 g of graphite (SG or G-waste-derived) was placed in a 2 L beaker, and 1 L of deionized water was added. The suspension (5 mg/mL) was treated for 60 min at 8000 rpm using a Ross high-shear mixer. The resulting solid was collected by gravity filtration and then dried for 16 h at 70 °C in an electric oven. The resulting materials were labeled LPE-SG and LPE-G-waste-derived, derived from SG or G-waste-derived, respectively.

#### 2.2.3. Synthesis of Graphene Oxides

GOs were prepared from SG, G-waste-derived, LPE-SG and LPE-G-waste-derived graphites using the modified Hummers method under carefully controlled reaction conditions. This method uses different amounts of graphite and KMnO_4_ (in a 1:3 ratio). In particular, 125 mL of concentrated H_2_SO_4_ were added to a 1 L beaker with magnetic stirring, then 15 g of KMnO_4_ was added to the beaker, and the mixture was cooled to 0 °C using an ice bath. Graphite was then added slowly in small doses. The solution was heated to 35 °C and stirred for 3 h. Afterwards, it was poured into a glass containing 500 mL of distilled water, the volume was raised to 1 L, and 20 mL of H_2_O_2_ (30%) was added and stirred for 5 more min. The suspension was washed by decantation using HCl and distilled water until a neutral pH. Once cleaned, the solid obtained was ultrasonicated in deionized water for 4 h. The sample was dried by freeze-drying. The resulting graphene oxides were labeled GO-SG, GO-G-waste-derived, GO-LPE-SG and GO-LPE-G-waste-derived, depending on the parent material used.

### 2.3. Synthesis of Amine-Functionalized Graphene Oxide

Amine-functionalized graphene oxides were prepared from GOs (GO-SG, GO-G-waste-derived, GO-LPE-SG and GO-LPE-G-waste-derived). In a typical experiment, 200 mg of graphene oxide are placed in a 40 mL vial, and 20 mL of hot ethanol is added. The vial is placed in a sand-containing beaker with hot ethanol, and the beaker is set on a hot plate to maintain the reaction temperature at 78 °C. The tip of a Cole-Parmer Instruments sonotrode (model CPX 130 PB with a CV-188 tip, Vernon Hills, IL, USA) is immersed halfway into the mixture, which is sonicated at 60% intensity for two minutes to disperse the solid. After this time, 200 μL of liquid n-octylamine (C8 amine) is added using a micropipette, and sonication is continued for 10 additional minutes. The mixture is transferred to a 200 mL beaker with the aid of ethanol to adjust the volume to 150 mL. After sedimentation, the supernatant is decanted, and the solid is treated with additional ethanol and stirred with the aid of a Vortex-Genie (Bohemia, NY, USA), allowed to sediment, and the supernatant removed once more, repeating the washing treatment a second time. The final decanted solid is filtered under vacuum using a Whatman grade 1 qualitative filter, then rinsed with 200 mL of hot ethanol followed by 500 mL of acetone. The solid was dried overnight at 70 °C in a vacuum electric oven. The products were labeled as Y-C8, where Y denotes the parent graphene oxide used (GO-SG, GO-LPE-SG, GO-G-waste-derived, GO-LPE-G-waste-derived) and C8 is n-octylamine.

### 2.4. Characterization Techniques

The structural and chemical characterization of the graphitic materials was performed by X-ray diffraction (XRD), Fourier transform infrared spectroscopy (FTIR), Raman spectroscopy, transmission electron microscopy (TEM), scanning electron microscopy (SEM), elemental analysis (EA), and thermogravimetric analysis (TGA). Detailed instrumental conditions and measurement parameters are provided in the Supplementary Information (Section S1).

## 3. Results

### 3.1. Characterization of Commercial Graphite and Graphites from Coke-like Wastes

**Figure 1:**
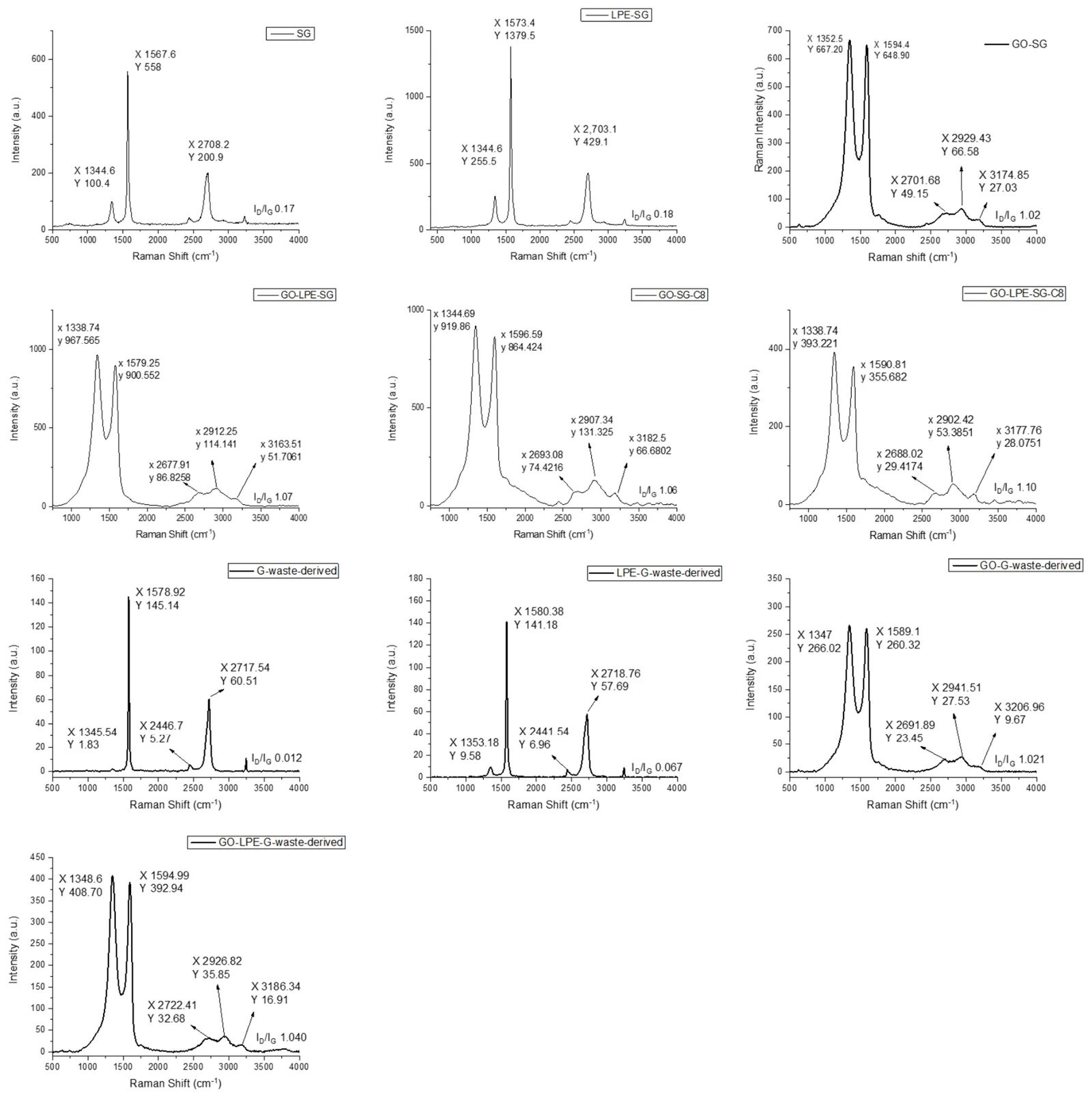
Raman spectra of SG, LPE-SG, GO-LPE-SG, GO-SG-C8, GO-LPE-SG-C8, G-waste-derived, LPE-G-waste-derived, GO-G-waste-derived, and GO-LPE-G-waste-derived samples. The characteristic D (~1350 cm^−1^) and G (~1580 cm^−1^) bands, associated with structural defects and the in-plane vibration of sp^2^-hybridized carbon atoms, respectively, are observed in all samples. Additionally, the second-order bands 2D (~2700 cm^−1^), 2D + G (~2930 cm^−1^), and 2D′ (~3200 cm^−1^) are identified. Variations in band intensity, position, and the I_D_/I_G_ ratio reflect the structural and electronic changes induced by liquid-phase exfoliation (LPE), oxidation, and octylamine functionalization.

The carbonaceous waste which intended to be valorized in this study, denoted as CkH, is a solid waste generated during the coke-making process for the production of metallurgical coke used as a feedstock for iron production in blast furnaces. This material typically accumulates in the upper inner section of the coking ovens during repeated operating cycles and must eventually be removed by mechanical scraping. CkH contains 11.3 wt.% ash, with the inorganic fraction consisting predominantly of Al (40–45 wt.%), Fe (39–42 wt.%), and Ca (13–15 wt.%), together with lower contents of Mg (1–2 wt.%) and Zn (0.5–1.5 wt.%), as determined by ICP-MS (see Materials and Methods section). On an ash-free basis, elemental analysis (Table 1) revealed a carbon content of 98.54 wt.%, with H, N, and S contents of 0.30, 0.34, and 0.82 wt.%, respectively. X-ray diffraction analysis of CkH (Table 1) showed an interlayer spacing of (d_002_ = 0.346) nm, with crystallite dimensions of Lc = 4.2 nm and La = 6.9 nm. Raman spectroscopy yielded an (I_D_/I_G_) ratio of 0.94 (see Figure 1), indicating the presence of a relatively defective and structurally heterogeneous carbon matrix.

To obtain a graphene oxide from this carbonaceous waste, CkH was subsequently subjected to a graphitization treatment (2800 °C), yielding G-waste-derived. After graphitization, with respect to those of CkH, the parameter d_002_ decreased to 0.336 nm, whereas Lc and La increased markedly to 35.0 and 56.7 nm, respectively (Table 1), indicating the development and growth of graphitic crystalline domains [39,40]. In parallel, the (I_D_/I_G_) ratio decreased substantially from 0.94 for CkH to 0.28 for G-waste-derived (see Figure 1), providing additional evidence of increased structural ordering during graphitization. This pronounced reduction in the defect-related Raman contribution, together with the increase in crystallite dimensions, suggests that the graphitization treatment promoted extensive structural reorganization of the carbon matrix [41,42]. We hypothesize that the mineral fraction originally present in CkH, particularly its Fe-containing component, may have contributed to this process by promoting carbon reorganization and graphitic ordering during the high-temperature treatment [43,44,45]. For comparison, a commercial graphite (SG) was used as a reference material (Table 1). SG exhibited the same d_002_ value as G-waste-derived (0.336 nm), but smaller crystallite dimensions, with Lc = 20.8 nm and La = 48.2 nm. Its elemental composition was 99.50 wt.% C, 0.42 wt.% H, 0.07 wt.% N, and 0.01 wt.% S. Thus, although G-waste-derived and SG display comparable interlayer spacings and highly carbonaceous compositions, G-waste-derived exhibits larger crystallite dimensions and a lower (I_D_/I_G_) ratio, indicating a more highly ordered graphitic structure according to these parameters. These two graphitic materials, G-waste-derived and SG, were subsequently employed as precursors for the preparation of graphene oxide directly or via the pretreatment via Liquid Phase Exfoliation. Raman spectra of the graphitized materials displayed the characteristic D, G and 2D bands of graphitic carbons. For SG, the D, G and 2D bands were located at 1344.6, 1567.6 and 2708.2 cm^−1^, respectively, while G-waste-derived exhibited bands at 1354.5, 1578.9 and 2717.5 cm^−1^ (Figure 1). Both materials showed low I_D_/I_G_ ratios (0.17 for SG and 0.12 for G-waste-derived), indicating a high degree of graphitic ordering and a relatively low defect density. The well-defined 2D bands further support the presence of an ordered graphitic structure. These results are consistent with the XRD analysis, which revealed the successful transformation of the carbonized precursors into highly graphitized materials.

### 3.2. Pretreatment of Graphitized Coke-like Waste (G-Waste-Derived) and Synthetic Graphite (SG)

**Figure 2:**
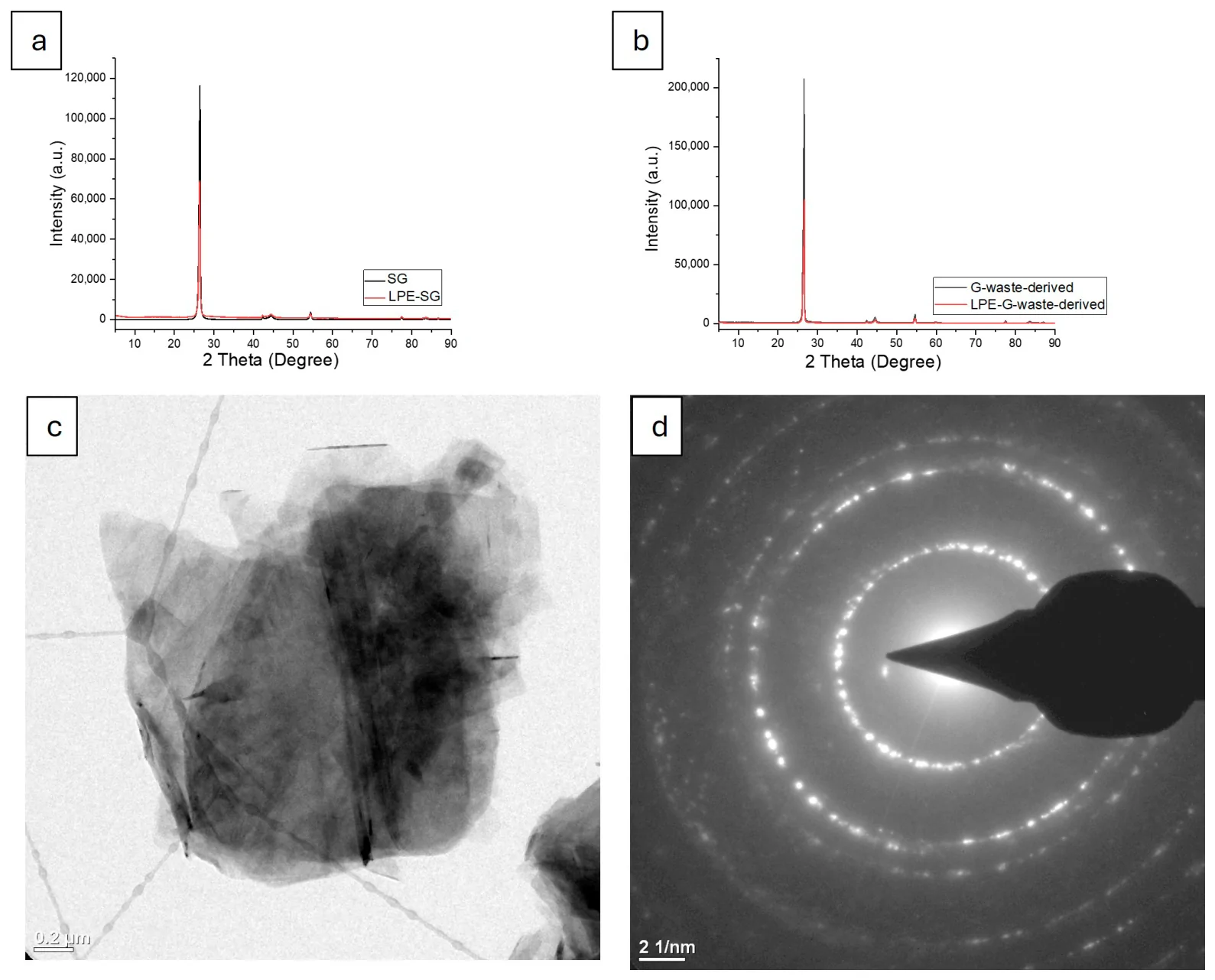
Structural characterization of graphite materials before and after liquid-phase exfoliation (LPE). (**a**) XRD patterns of SG and LPE-SG. (**b**) XRD patterns of G-waste-derived and LPE-G-waste-derived. (**c**) TEM image of LPE-SG. (**d**) Corresponding selected-area electron diffraction (SAED) pattern of LPE-SG showing the preservation of the graphitic crystalline structure after the exfoliation treatment.

Authors recently reported that the aqueous liquid-phase exfoliation (LPE) of graphite using water as the sole liquid medium for high-shear mixing is an effective route for producing few-layer graphene nanoplatelets [46]. This represents an attractive alternative to conventional LPE approaches, which typically employ high-boiling organic solvents such as N-methyl-2-pyrrolidone (NMP) or dimethylformamide (DMF) to stabilize exfoliated graphene sheets in suspension [47,48,49,50]. Based on these results, a mild aqueous exfoliation treatment was applied in the present work to the graphite derived from the coke-like waste (G-waste-derived). For comparison, the same procedure was also applied to commercial graphite (SG). In the present study, this approach was adapted as a structural pretreatment of G-waste-derived and SG prior to its oxidation by the Hummers method, with the aim of modifying the accessibility and dispersion of the graphitic domains before the subsequent oxidation step. The resulting aqueous-exfoliated materials, denoted as LPE-G-waste-derived and LPE-SG, respectively, were characterized by XRD (Table 1, Figure 2).

As shown in Figure 2a,b, the aqueous LPE treatment modified the (002) reflection of both graphitic precursors, with a more pronounced attenuation observed for G-waste-derived under the same experimental conditions. The more pronounced decrease in the (002) intensity for G-waste-derived indicates that LPE mainly disrupts the coherent stacking of graphene layers in this precursor, affecting the crystallite thickness to a greater extent than the lateral dimension of the graphitic domains. Nevertheless, this attenuation of the (002) reflection is consistent with the structural changes associated with aqueous high-shear exfoliation in aqueous media reported by Cuara et al. [46]. The interlayer spacing d_002_ remained essentially unchanged, decreasing from 0.336 to 0.335 nm for both G-waste-derived and SG, indicating that the characteristic graphitic interlayer distance was largely preserved after the aqueous treatment. This behavior demonstrates that the LPE treatment does not significantly alter the graphitic lattice itself, but rather reduces the number of graphene layers contributing coherently to diffraction. The crystallite thickness (Lc), which describes the dimension perpendicular to the graphene layers and is therefore related to the number of coherently stacked layers, decreased from 35.0 to 29.7 nm for G-waste-derived, whereas the lateral crystallite dimension (La), associated with the in-plane extension of the graphitic domains, decreased from 56.7 to 52.4 nm. The estimated number of coherently stacked graphitic layers (N) was subsequently calculated according to:

N = (Lc/d_002_) + 1

where d_002_ is the interlayer spacing obtained from Bragg’s law. This relationship has been widely used for estimating the average number of stacked graphene layers in graphitic materials from XRD-derived structural parameters [51,52]. As observed there is a decreased from approximately 105 for G-waste-derived to 90 for LPE-G-waste-derived. In contrast, SG showed a smaller reduction in Lc, from 20.8 to 18.7 nm, while La decreased more substantially, from 48.2 to 34.4 nm. Accordingly, the estimated number of coherently stacked graphitic layers decreased from approximately 63 for SG to 57 for LPE-SG. These differences indicate that the aqueous treatment does not affect the two graphitic precursors in an identical manner. For G-waste-derived, the greater change in Lc relative to La indicates that LPE predominantly modified the stacking direction, reducing the crystallite thickness while largely preserving its lateral dimensions. In contrast, the more pronounced reduction in La observed for SG indicates a greater modification of the lateral extension of its graphitic domains, while the change in Lc was comparatively limited. Thus, LPE primarily affected the stacking dimension of G-waste-derived, whereas its effect on SG was more pronounced in the lateral dimension, demonstrating that the structural response to the aqueous treatment depends on the nature of the graphitic precursor [32].

To evaluate the reproducibility of the LPE treatment, independent batches were prepared under identical experimental conditions and the intensity of the graphite (002) diffraction peak was analyzed statistically (Figure S1, Supplementary Material). The resulting box-plot distributions show that the decrease in the (002) peak intensity with increasing impeller rotation speed is consistently reproduced among independent batches. While slightly larger dispersion was observed at 6000 and 8000 rpm and an isolated outlier appeared at 10,000 rpm, the overall trend remained unchanged. These results confirm the reproducibility of the structural modifications induced by LPE and support the use of 8000 rpm as a suitable operating condition, providing substantial structural modification while maintaining good reproducibility.

Raman spectroscopy was used to evaluate the effect of the aqueous liquid-phase exfoliation treatment on the graphitic structure (Figure 1). Following LPE, only minor changes were observed in the positions of the D, G and 2D bands for both SG and G-waste-derived. Likewise, the ID/IG ratios remained low, changing from 0.17 to 0.18 for SG and from 0.12 to 0.07 for G-waste-derived. These limited variations indicate that the exfoliation treatment introduces little additional structural disorder and largely preserves the graphitic framework. The Raman results therefore suggest that the aqueous LPE treatment mainly promotes physical delamination and rearrangement of stacked graphitic domains rather than significant chemical or structural modification of the carbon lattice, in agreement with the XRD observations.

TEM analysis further confirmed the structural modifications induced by the LPE treatment (Figure 2c,d, representative of the exfoliation process). The commercial graphite precursor (SG) consisted of particles with nominal lateral dimensions below 20 μm, whereas TEM observations of the exfoliated material (LPE-SG) revealed thin graphitic flakes with lateral sizes typically ranging from 1 to 2 μm. The high transparency of these flakes, compared with the electron-opaque precursor particles, suggests a substantial reduction in the number of stacked graphitic layers after the LPE treatment, a behavior commonly reported for liquid-phase exfoliated graphitic materials [35,37]. Furthermore, the well-defined diffraction features observed in the corresponding SAED pattern confirm that the graphitic crystalline structure was largely preserved despite the reduction in particle size and partial delamination of the crystallites. These structural modifications are expected to facilitate the penetration of oxidizing species into the graphitic domains during the subsequent Hummers oxidation process by increasing the exposure of graphitic surfaces and reducing mass-transfer limitations within the crystallites, as previously proposed for pre-expanded graphitic materials [34].

**Figure 3:**
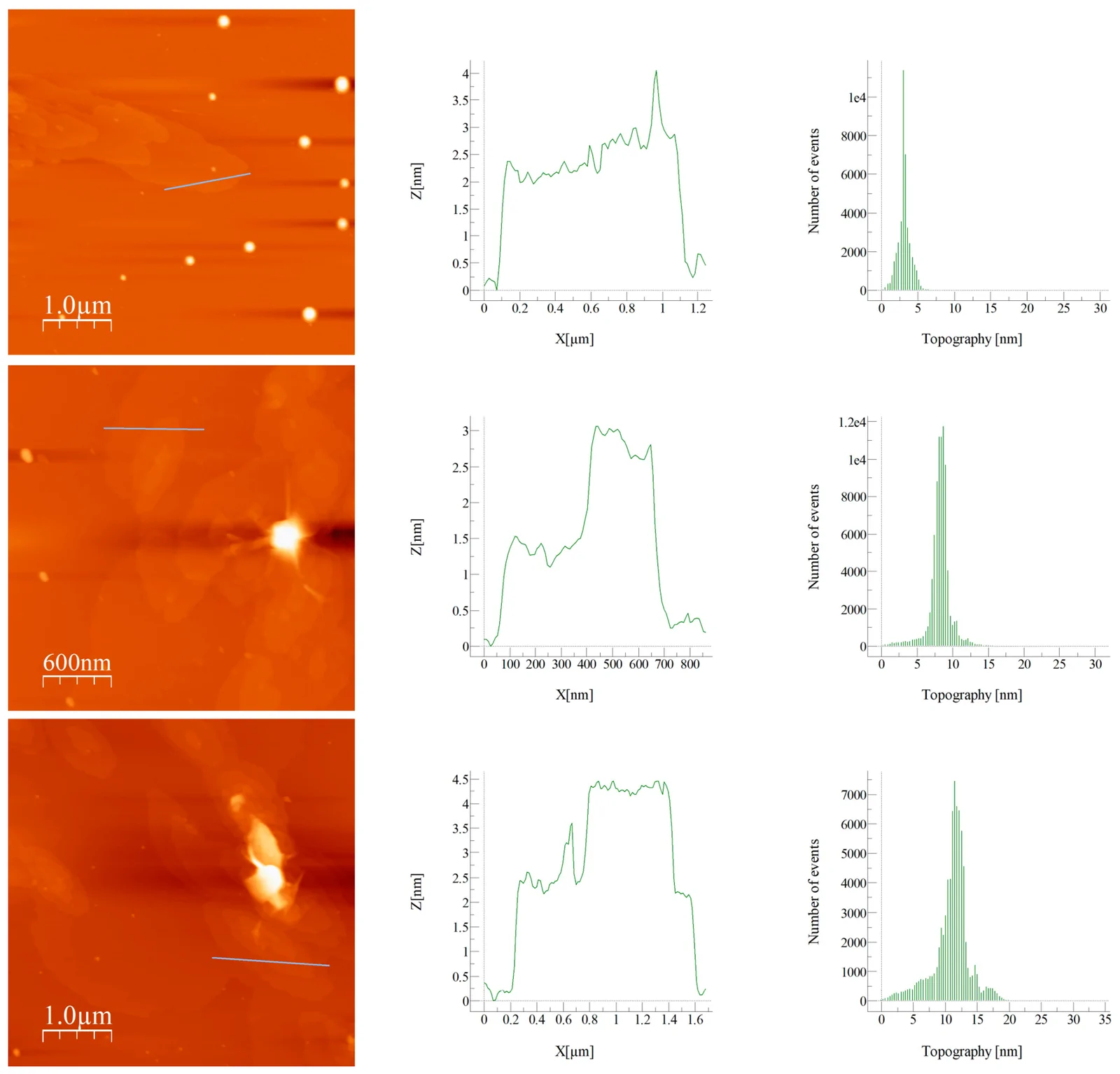
AFM image (**left**), profiles (**center**) and histograms (**right**) of LPE-SG The blue line in the AFM image indicates the location where the AFM topography profile was acquired.

To directly verify whether the aqueous treatment induced liquid-phase exfoliation rather than simple particle fragmentation, atomic force microscopy (AFM) was performed on the representative LPE-SG sample (Figure 3). The AFM topographic images revealed laterally extended lamellar structures with a well-defined sheet-like morphology characteristic of graphenic flakes. Representative height profiles showed thicknesses ranging from approximately 1.5 to 4 nm, while the corresponding thickness distribution was mainly comprised between 1 and 15 nm. Particle fragmentation alone would primarily decrease the lateral dimensions of the particles without necessarily reducing the number of stacked graphene layers. In contrast, the observation of laterally extended flakes with nanometric thicknesses indicates a reduction in graphitic layer stacking and supports the occurrence of partial exfoliation during the LPE treatment. These observations are also consistent with the XRD and TEM results discussed below.

Furthermore, FTIR analysis of the untreated and LPE-treated materials (Figure S2, Supplementary Material) revealed no significant differences in the characteristic oxygen-containing functional group bands, indicating that the aqueous LPE treatment primarily induces structural modifications while largely preserving the original surface chemistry. These findings, together with the AFM, TEM, and XRD results, indicate that the structural modifications induced by the LPE treatment are retained after drying, suggesting that any restacking occurring during water removal is limited.

### 3.3. Oxidation of Graphitized Coke-like Waste and Commercial Graphite, with and Without LPE Pretreatment

Graphene oxide samples were prepared from G-waste-derived, SG and their corresponding liquid-phase exfoliated counterparts (LPE-G-waste-derived and LPE-SG) using a modified Hummers oxidation method. This Hummers oxidation protocol was applied in a controlled manner taking into account the presence of inorganic impurities in the graphitized waste. Since the acid treatment removes the majority of metallic species, their contribution during the subsequent amination step is expected to be negligible.

**Figure 4:**
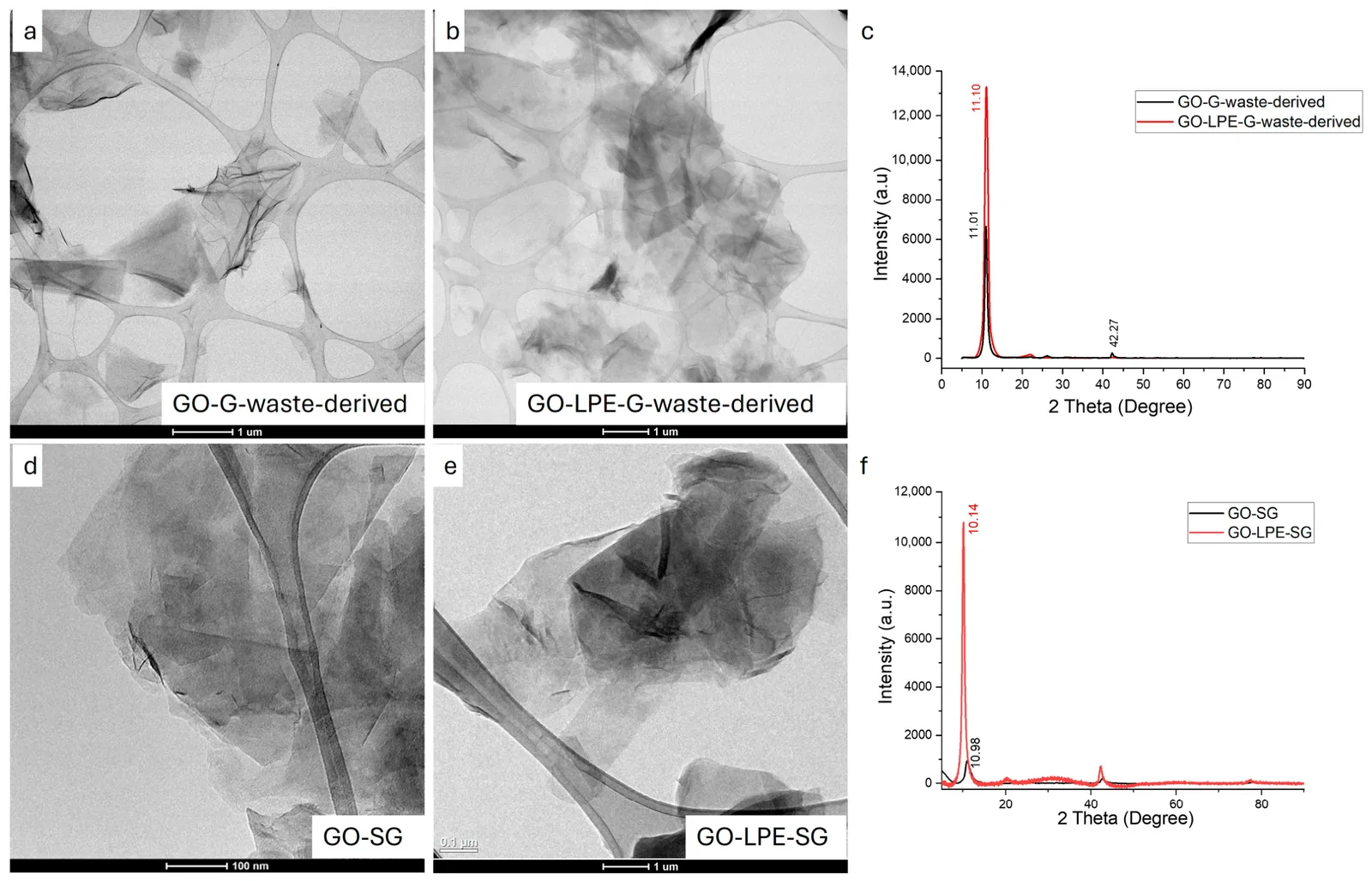
Structural characterization of graphene oxides obtained from graphitized coke-derived graphite (G-waste-derived) and synthetic graphite (SG). (**a**) TEM image of GO-G-waste-derived. (**b**) TEM image of GO-LPE-G-waste-derived. (**c**) XRD patterns of GO-G-waste-derived and GO-LPE-G-waste-derived. (**d**) TEM image of GO-SG. (**e**) TEM image of GO-LPE-SG. (**f**) XRD patterns of GO-SG and GO-LPE-SG.

Representative TEM images of the GOs obtained are shown in Figure 4a,b,d,e. Regardless of the precursor employed, all samples exhibit the characteristic thin, wrinkled and partially transparent sheet-like morphology commonly associated with graphene oxide [5,53]. These observations confirm that both the commercial graphite (SG GOs) and the graphitized coke-like waste (G-waste-derived GOs) can be successfully transformed into graphene oxide through the modified Hummers oxidation process. The ability to obtain well-developed graphene oxide structures from G-waste-derived and LPE-G-waste-derived is particularly relevant, as it demonstrates the potential of this industrial waste-derived material as an alternative feedstock for the production of functionalized graphene materials. Furthermore, the absence of major morphological differences between the LPE-treated and untreated samples suggests that any structural changes induced by the exfoliation pretreatment occur primarily at the level of layer organization and stacking rather than through large-scale morphological modifications.

This interpretation is supported by XRD analysis. All oxidized materials displayed in the XRD spectra (Figure 4c for the G-waste-derived samples and Figure 4f for the SG-derived samples) the characteristic graphene oxide (001) reflection at approximately 10–11° (2θ), confirming the successful conversion of the graphitic precursors into graphene oxide [5]. Simultaneously, the characteristic graphite (002) reflection located at approximately 26° (2θ) in the parent materials almost completely disappeared after oxidation, indicating the disruption of the original graphitic stacking and the formation of oxidized layered structures [23]. The most notable feature was the marked increase in the intensity of the (001) reflection after the LPE pretreatment for both graphite precursors. This behavior suggests the formation of oxidized layers with a more uniform interlayer spacing and a higher degree of structural coherence. The SG-derived materials exhibited a noticeable shift in the (001) reflection from 10.98° to 10.14° after LPE treatment, corresponding to an increase in the average interlayer spacing. Such behavior suggests that the exfoliation pretreatment facilitated the penetration of oxidizing species into the graphitic structure, leading to a greater expansion of the oxidized layers [54]. In contrast, the position of the (001) reflection remained essentially unchanged for the G-waste-derived materials (11.01–11.10°), indicating that the interlayer spacing was only slightly affected by the exfoliation pretreatment. Nevertheless, the substantial increase in diffraction intensity observed for GO-LPE-G-waste-derived suggests that LPE promoted a more homogeneous organization of the oxidized layers [32]. Therefore, although the effect of LPE on the interlayer spacing differs according to the graphite precursor, the treatment consistently produces graphene oxides characterized by a more defined layered structure.

**Figure 5:**
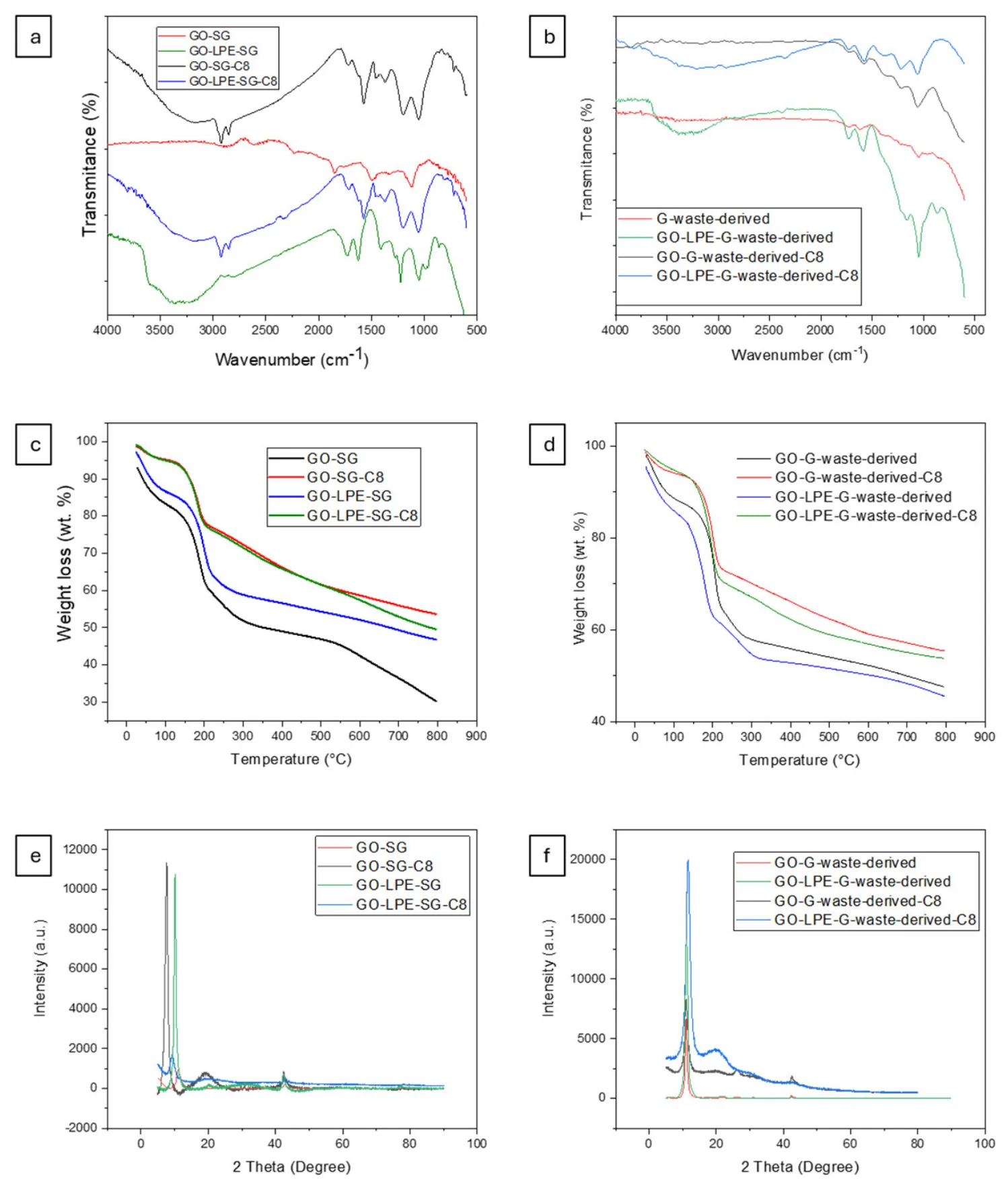
Structural, spectroscopic and thermal characterization of graphene oxides and their corresponding amine-functionalized derivatives. (**a**) FTIR spectra of GO-SG, GO-LPE-SG, GO-SG-C8 and GO-LPE-SG-C8. (**b**) FTIR spectra of GO-G-waste-derived, GO-LPE-G-waste-derived, GO-G-waste-derived-C8 and GO-LPE-G-waste-derived-C8. (**c**) TG curves of GO-SG, GO-LPE-SG, GO-SG-C8 and GO-LPE-SG-C8. (**d**) TG curves of GO-G-waste-derived, GO-LPE-G-waste-derived, GO-G-waste-derived-C8 and GO-LPE-G-waste-derived-C8. (**e**) XRD patterns of GO-SG, GO-LPE-SG, GO-SG-C8 and GO-LPE-SG-C8. (**f**) XRD patterns of GO-G-waste-derived, GO-LPE-G-waste-derived, GO-G-waste-derived-C8 and GO-LPE-G-waste-derived-C8.

FTIR spectroscopy was used to investigate the effect of the LPE pretreatment on the oxygen-containing functionalities generated during oxidation (Figure 5a,b). All graphene oxide samples exhibited the characteristic bands of oxidized graphitic materials, including the carbonyl stretching vibration at approximately 1725 cm^−1^, the aromatic skeletal vibration at 1617 cm^−1^, and the C–O/C–O–C bands located between approximately 1230 and 1040 cm^−1^, confirming the successful oxidation of both graphitic precursors [5,23].

For both G-waste-derived- and SG-derived materials, the LPE-treated samples exhibited more intense and better-defined bands in the 1230–1040 cm^−1^ region than their non-exfoliated counterparts. These absorptions are commonly associated with C–O and C–O–C functionalities generated during oxidation [22,54]. In contrast, the changes observed in the carbonyl band at 1725 cm^−1^ were comparatively less pronounced. This behavior suggests that the LPE pretreatment modifies the distribution of oxygen-containing functionalities generated during the oxidation process.

**Figure 6:**
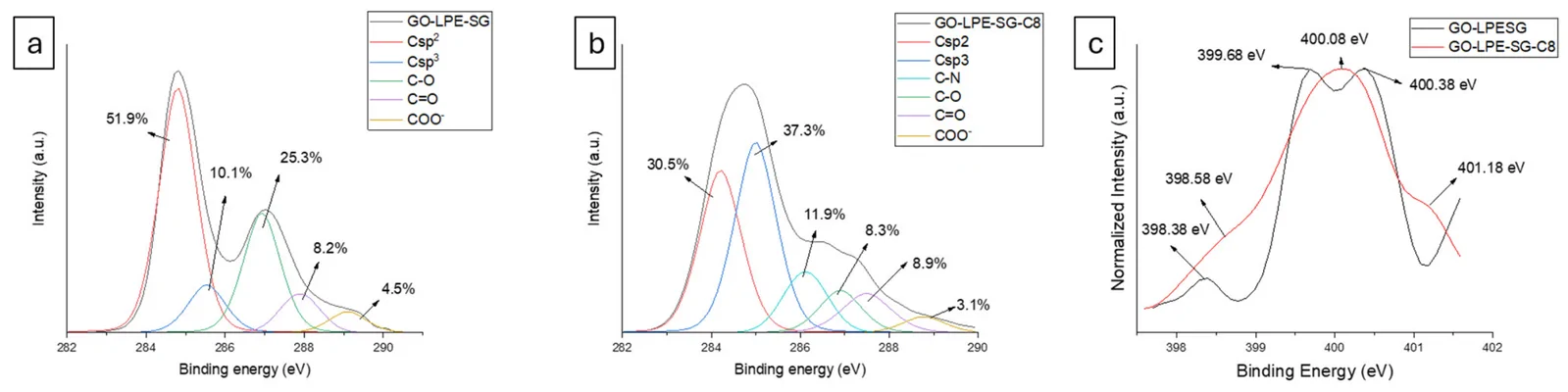
X-ray photoelectron spectroscopy (XPS) characterization of GO-LPE-SG and GO-LPE-SG-C8. (**a**) High-resolution C1s spectrum of GO-LPE-SG. (**b**) High-resolution C1s spectrum of GO-LPE-SG-C8 (**c**) High-resolution N1s spectra of GO-LPE-SG and GO-LPE-SG-C8.

To provide a quantitative assessment of the surface chemical composition, XPS analysis (Figure 6) was performed on GO-LPE-SG. Deconvolution of the C1s spectrum revealed contributions of 51.9% for C=C, 10.1% for C-C, 25.3% for C-O, 8.2% for C=O and 4.5% for O-C=O species. The substantial contribution of oxygen-containing functionalities (C-O, C=O and O-C=O) confirms the successful oxidation of the graphitic material and complements the qualitative information obtained from FTIR spectroscopy.

Raman spectroscopy provided additional evidence of the structural changes induced during the oxidation process (Figure 1). In both SG- and G-waste-derived materials, oxidation produced a marked increase in the *I*_D_/*I*_G_ ratio from values below 0.2 for the parent graphites to approximately 1.0 for all graphene oxides, indicating the generation of defects and the disruption of the extended sp^2^ carbon network. Simultaneously, the G band shifted towards higher wavenumbers (1579–1595 cm^−1^), consistent with the introduction of oxygen-containing functionalities and the modification of the electronic structure of the graphitic lattice.

In addition to the first-order D and G bands, all graphene oxide samples exhibited a well-defined second-order Raman region composed of three contributions centred at approximately 2680–2720 cm^−1^, 2900–2940 cm^−1^ and 3160–3210 cm^−1^, assigned to the 2D, D+G and 2D’ bands, respectively. The presence of the combination D+G band and the 2D’ contribution is commonly observed in defective graphitic materials and reflects the increased disorder introduced during oxidation. Although these second-order features remained detectable, their relative broadening and lower definition compared with the parent graphites are consistent with the loss of long-range structural order resulting from the Hummers oxidation process.

Overall, the Raman results corroborate the successful oxidation of both graphite precursors and are fully consistent with the structural modifications evidenced by XRD, FTIR and XPS.

Thermogravimetric analysis provides complementary information regarding the oxygen-containing functionalities generated during oxidation (Figure 5c,d). All graphene oxide samples exhibited the characteristic thermal behavior of graphene oxide, including an initial mass loss below approximately 120 °C associated with the removal of physically adsorbed water, followed by a major decomposition step between approximately 150 and 300 °C, commonly attributed to the thermal decomposition of labile oxygen-containing functionalities such as epoxides, hydroxyls and other oxygenated surface groups [5,23]. A more gradual mass loss extending from approximately 300 to 800 °C was also observed for all samples and is generally associated with the decomposition of more stable oxygen functionalities together with the progressive degradation of the carbon framework [5].

For the G-waste-derived materials, GO-LPE-G-waste-derived exhibited a slightly greater mass loss than GO-G-waste-derived throughout the decomposition process. This behavior is consistent with the FTIR results, which showed more intense and better-defined oxygen-related bands in the LPE-derived material, particularly in the 1230–1040 cm^−1^ region associated with C–O and C–O–C functionalities. These observations suggest that the exfoliation pretreatment modifies the oxidation behavior of the highly crystalline G-waste-derived precursor, promoting a larger population and/or a different distribution of oxygen-containing functionalities.

A similar tendency was observed for the SG-derived materials. Although the thermogravimetric profiles of GO-SG and GO-LPE-SG remained broadly comparable, the LPE-derived sample also exhibited a slightly higher mass loss, consistent with the enhanced oxygen-related absorptions observed by FTIR. In agreement with previous studies on graphite oxidation, these results indicate that modifications in the precursor structure influence the nature and distribution of the oxygen functionalities generated during the oxidation process [32].

### 3.4. Amination of GOs

The effect of the amination reaction on the structural organization of the graphene oxides was investigated by XRD. As shown in Figure 5e,f, all aminated materials retained the characteristic low-angle diffraction peak associated with the layered graphene oxide structure, indicating that the graphenic framework remained largely preserved after functionalization. However, noticeable shifts in the diffraction maxima were observed after reaction with n-octylamine. The SG-derived materials exhibited the largest changes, with the diffraction maximum shifting from 10.98° for GO-SG to 7.61° for GO-SG-C8, while GO-LPE-SG shifted from 10.14° to 9.35°. Similar, although less pronounced, changes were observed for the G-waste-derived materials, with diffraction maxima located at 11.51° and 10.98° for GO-G-waste-derived-C8 and GO-LPEG-waste-derived-C8, respectively. These shifts indicate modifications in the average interlayer spacing following functionalization and are consistent with the structural rearrangements commonly reported during amine functionalization of graphene oxide through epoxide ring-opening reactions [7,24,25]. Importantly, the structural differences established during the oxidation stage were largely preserved after amination, suggesting that the organization of the parent graphene oxide continues to influence the final structure of the functionalized materials.

FTIR spectroscopy was used to investigate the chemical modifications induced by the amination reaction (Figure 5a,b). Relative to the corresponding graphene oxides, all aminated materials exhibited noticeable changes in the oxygen-related absorption bands, particularly those located at approximately 1725 cm^−1^ and in the 1230–1040 cm^−1^ region associated with C–O and C–O–C functionalities. These groups are commonly recognized as the main reactive sites involved in the functionalization of graphene oxide with primary amines [7,24,25].

The spectral modifications were observed for both G-waste-derived- and SG-derived materials, although they were more pronounced for the LPE-derived samples. Compared with their corresponding graphene oxides, GO-LPE-G-waste-derived-C8 and GO-LPE-SG-C8 exhibited a greater alteration of the oxygen-related bands, suggesting a higher participation of the oxygen-containing functionalities generated during oxidation. This behavior is consistent with the FTIR results of the parent graphene oxides, where the LPE-derived materials displayed stronger C–O and C–O–C absorptions prior to amination.

Although FTIR does not allow a quantitative determination of the grafted amine content, the observed spectral changes clearly indicate that oxygen-containing groups participated in the reaction. Therefore, the results suggest that the LPE pretreatment influences the accessibility and chemical environment of the oxygen functionalities available for subsequent covalent modification.

To further investigate the chemical modifications induced by the amination treatment, XPS analysis was performed on GO-LPE-SG and GO-LPE-SG-C8 (Figure 6a,b). The survey spectra revealed an increase in nitrogen content from 1.8 at.% for GO-LPE-SG to 2.6 at.% after reaction with n-octylamine, confirming the successful incorporation of nitrogen-containing species. Consistent with this observation, the high-resolution C 1s spectrum of the aminated material (Figure 6b) required the introduction of an additional component assigned to C-N environments, centered between the C-C and C-O contributions. The incorporation of this contribution significantly improved the fitting quality and constitutes further evidence of the chemical modification of the graphene oxide surface.

A comparison of the C 1s deconvolution profiles showed that the relative contribution of oxygen-containing functionalities decreased after amination. In particular, the C-O component decreased from 25.3% in GO-LPE-SG to 8.3% in GO-LPE-SG-C8. However, this decrease should be interpreted with caution because the newly introduced C-N contribution (11.9%) partially overlaps the binding-energy region located between the C-C and C-O signals. Therefore, part of the apparent reduction may arise from the redistribution of spectral intensity associated with the improved fitting model rather than exclusively from the consumption of oxygen functionalities.

Nevertheless, the increase in nitrogen content after octylamine treatment, together with the appearance of a C-N contribution in the C 1s spectrum, supports the successful incorporation of nitrogen-containing functionalities onto the graphene oxide surface. In agreement with this observation, the N 1s spectrum of GO-LPE-SG-C8 exhibited a broad signal centered at approximately 400.1 eV (Figure 6c), consistent with amine-containing species, whereas only a weak nitrogen signal was detected in the precursor material. The nitrogen content increased approximately 1 at.% after functionalization, further supporting the incorporation of amine-derived groups following the amination treatment.

Raman spectroscopy revealed only minor changes after amination (Figure 1). The *I*_D_/*I*_G_ ratios of the aminated materials remained very similar to those of their corresponding graphene oxides, changing from 1.02 to 1.06 for GO-SG, from 1.07 to 1.10 for GO-LPE-SG, while GO-HG and GO-LPE-HG exhibited comparable values. Likewise, only small shifts were observed in the D and G band positions after reaction with *n*-octylamine.

The second-order Raman region was also preserved after functionalization. The aminated samples continued to exhibit the characteristic 2D, D+G and 2D’ contributions, located at approximately 2688–2693 cm^−1^, 2902–2907 cm^−1^ and 3178–3183 cm^−1^, respectively. The persistence of these bands, together with the limited variation in the *I*_D_/*I*_G_ ratios, indicates that the carbon framework remained largely unaffected during the amination treatment.

These observations suggest that the reaction with *n*-octylamine primarily involves surface chemical modification of the oxygen-containing functionalities rather than extensive disruption of the graphenic lattice. This interpretation is fully consistent with the FTIR and XPS results, which demonstrate the incorporation of nitrogen-containing species while preserving the overall structural integrity of the graphene oxide sheets.

Thermogravimetric analysis was employed to further evaluate the structural modifications induced by the amination treatment (Figure 5c,d). All aminated materials exhibited the characteristic thermal behavior of alkylamine-functionalized graphene oxides, including an initial mass loss below approximately 120 °C associated with the removal of residual adsorbed species, followed by a broad decomposition region extending from approximately 200 to 600 °C. This thermal event is generally associated with the decomposition of residual oxygen-containing functionalities together with the degradation of surface species generated during the reaction with primary amines [5,24]. The overall TG profiles of the aminated samples remain relatively similar, suggesting that the amination treatment does not drastically alter the bulk thermal stability of the graphene oxide framework. This behavior indicates that the thermal decomposition process involves surface functionalities with different thermal environments and/or stability distributions. These observations are consistent with the FTIR results, which showed a greater modification of the oxygen-related bands after amination of the LPE-derived graphene oxides. Taken together, the FTIR and TG results suggest that the oxygen-containing functionalities generated after the LPE pretreatment participate differently in the reaction with n-octylamine. Although thermogravimetric analysis does not allow the amount of grafted amine to be directly quantified, the broader decomposition profiles observed for the LPE-derived materials indicate that the exfoliation pretreatment modifies the accessibility and chemical environment of the reactive oxygen functionalities involved in the functionalization process.

**Figure 7:**
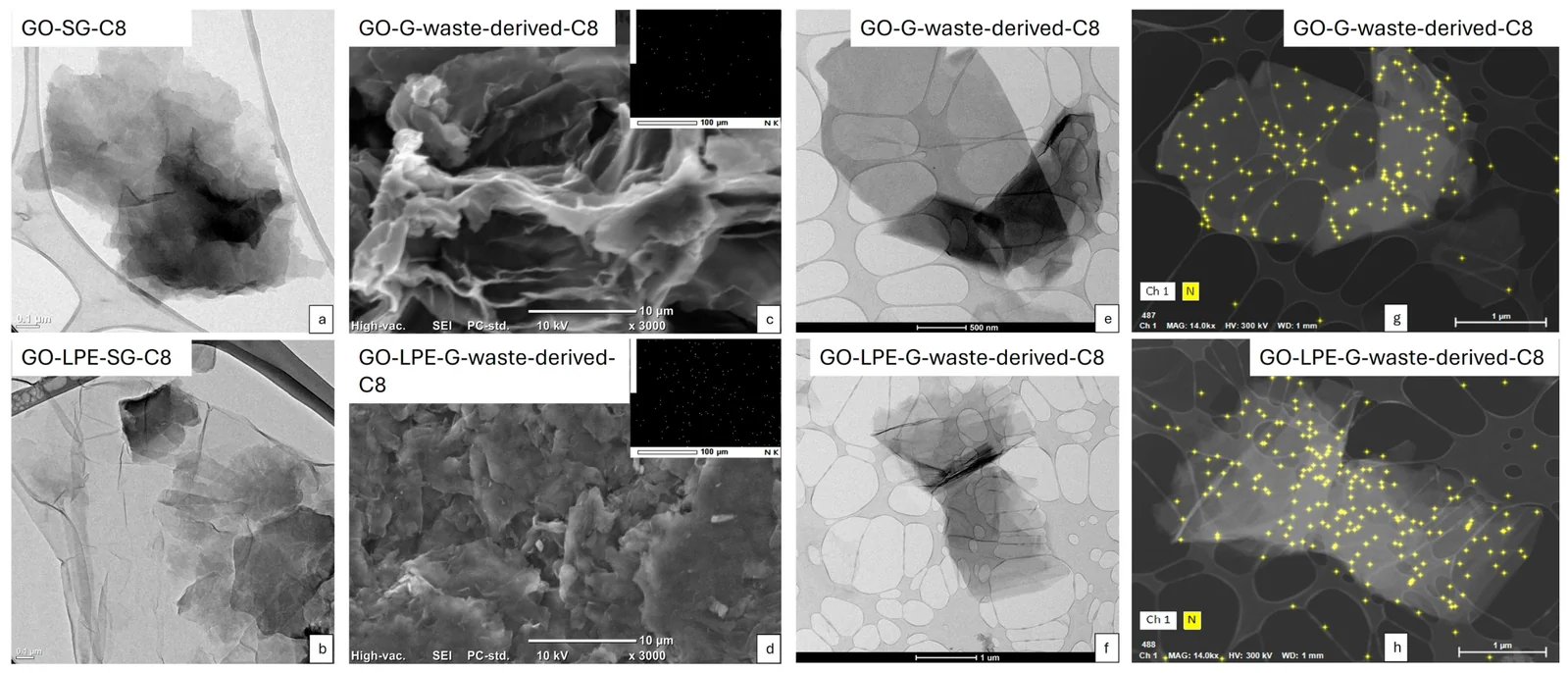
Morphological characterization of the amine-functionalized graphene oxides. (**a**) TEM image of GO-SG-C8. (**b**) TEM image of GO-LPE-SG-C8. (**c**) SEM image of GO-G-waste-derived-C8 and corresponding EDS elemental mapping of nitrogen (inset). (**d**) SEM image of GO-LPE-G-waste-derived-C8 and corresponding EDS elemental mapping of nitrogen (inset). (**e**) TEM image of GO-G-waste-derived-C8. (**f**) TEM image of GO-LPE-G-waste-derived-C8. (**g**) EDS elemental mapping of nitrogen for GO-LPE-G-waste-derived-C8. (**h**) EDS elemental mapping of nitrogen for GO-G-waste-derived-C8.

The morphology of the amine-functionalized graphene oxides was examined by electron microscopy (Figure 7). Representative TEM images of the SG-derived materials (Figure 7a,b) reveal thin and transparent graphenic sheets, characteristic of exfoliated graphene oxide structures. Both GO-SG-C8 and GO-LPE-SG-C8 retained their layered morphology after amination, indicating that the functionalization process did not significantly alter the graphene oxide framework. Additional TEM analysis of the G-waste-derived samples (Figure 7e,f) confirmed the presence of thin graphenic sheets with preserved layered morphology after amination. More importantly, the corresponding TEM-EDS nitrogen elemental maps (Figure 7g,h) revealed a homogeneous distribution of nitrogen across the graphene sheets, providing direct evidence of the effective incorporation of nitrogen-containing functionalities throughout the material.

The G-waste-derived materials were characterized by SEM (Figure 5c,d) and exhibited the characteristic folded and interconnected lamellar morphology of graphene-based materials. No major morphological differences were observed between GO-G-waste-derived-C8 and GO-LPE-G-waste-derived-C8, suggesting that the amination treatment preserved the overall microstructure of the materials. The nitrogen distribution observed by TEM-EDS is consistent with the homogeneous functionalization inferred from the SEM-EDS analysis, while providing higher spatial-resolution information at the individual-sheet level.

These observations indicate that the ultrasound-assisted amination process produced a relatively uniform incorporation of nitrogen-containing functionalities regardless of the precursor employed or the application of the LPE pretreatment.

Particularly relevant is the fact that the graphene oxides derived from the graphitized coke residue exhibited morphological characteristics and nitrogen distributions comparable to those observed for the commercial graphite-derived materials. This behavior demonstrates that the graphitized waste can be effectively transformed into functionalized graphene materials without compromising the homogeneity of the amination process, highlighting its potential as a low-cost and sustainable alternative precursor for the production of value-added nitrogen-functionalized graphenes.

## 4. Conclusions

Graphene oxide (GO) and n-octylamine-functionalized graphene oxide were successfully synthesized from graphitized coke-derived residue and compared with analogous materials prepared from commercial synthetic graphite. The results demonstrate that graphitized coke waste is a suitable precursor for the production of graphene-based materials, yielding GO with structural, morphological, and chemical characteristics comparable to those obtained from conventional graphitic sources.

Aqueous liquid-phase exfoliation (LPE) modified the structural organization of the graphitic precursors, reducing particle size and stacking while preserving graphitic crystallinity. After oxidation, LPE-derived materials showed enhanced development of oxygen-containing functionalities, as evidenced by FTIR, TG, and XPS analyses.

Subsequent functionalization with n-octylamine resulted in detectable structural, spectroscopic, and thermal changes, together with the incorporation of nitrogen-containing species confirmed by XPS and TEM-EDS mapping. These findings indicate that the LPE pretreatment influences the accessibility and reactivity of surface functional groups toward covalent modification.

Overall, the combination of graphitized coke waste and aqueous LPE provides a viable and sustainable route for producing functionalized graphene-based materials, supporting the valorization of industrial carbon residues into higher-value nanomaterials.

## Figures and Tables

**Table 1:** XRD and elemental parameters of raw materials SG, CkH and G-waste-derived and LPE graphites (LPE-SG and LPE-G-waste-derived).

Sample	XRD (nm)			Elemental Analysis (wt.%) ^a^			
	d_002_	Lc	La	C	H	N	S
SG	0.336	20.8	48.2	99.50	0.42	0.07	0.01
CkH	0.346	4.2	6.9	98.54	0.30	0.34	0.82
G-waste-derived	0.336	35.0	56.7	99.60	0.30	0.09	0.01
LPE-SG	0.335	18.7	34.4	99.59	0.35	0.05	0.01
LPE-G-waste-derived	0.335	29.7	52.4	99.61	0.29	0.09	0.01

^a^ = ash-free basis.

## Data Availability

The original contributions presented in this study are included in the article/Supplementary Material. Further inquiries can be directed to the corresponding authors.

## Supplementary Materials

The following supporting information can be downloaded at: https://www.mdpi.com/article/10.3390/nano16211434/s1, Section S1: Details of characterization techniques; Section S2: Analysis of reproducibility; Figure S1: Boxplot representation of the X-ray intensity measured for samples obtained at different centrifugation speeds (4000, 6000, 8000 and 10,000 rpm). The boxes represent the interquartile range (25–75%), the horizontal line inside each box corresponds to the median, the square symbol indicates the mean value, whiskers extend to values within 1.5 times the interquartile range (IQR), and diamonds represent outliers; Section S3: FTIR spectra of the untreated and LPE-treated graphitic materials; Figure S2: FTIR spectra of the untreated and LPE-treated graphitic materials.
