# Supplementary material for: From Graphitized Coke Waste to Aminated Graphene Oxide: Enhancing Reactive Oxygen Functionalities Through Liquid-Phase Exfoliation Pretreatment
Source: Nanomaterials (Basel). 2026 Nov 6;16(21):1434. doi: 10.3390/nano16211434 (PMC13765151; doi:10.3390/nano16211434)
Supplement: Supplementary file 1 [file nanomaterials-16-01434-s001.zip › nanomaterials-4632480-supplementary.pdf]

Supplementary Material

From Graphitized Coke Waste to Aminated Graphene Oxide: Enhancing Reactive Oxygen Functionalities through Liquid-Phase Exfoliation Pretreatment.

Maiby Valle Orta[1], Edgar Cuara [1], Alfonso Mercado[1], Enrique Díaz Barriga Castro[1], Pablo González Morones[1], Marcos Granda[2], Diego Martínez[3], Patricia Álvarez [2]*and Uriel Sierra[1,]*

[1] Laboratorio Nacional de Materiales Grafénicos, Centro de Investigación en Química Aplicada. Blvd. Enrique Reyna Hermosillo 140, San José de los Cerritos, Coahuila, México, 25117.
[2] Instituto de Ciencia y Tecnología del Carbono INCAR-CSIC. C/ Francisco Pintado Fe, 26. 33011, Oviedo, España.
[3] Centro de Investigación en Geociencias Aplicadas, Nueva Rosita, 26830, Coahuila, México.
* Patricia Álvarez: par@incar.csic.es
* Uriel Sierra: uriel.sierra@ciqa.edu.mx

S1.- Details of characterization techniques.

Elemental analysis (EA): The oxygen content of the samples was determined directly in a LECO-TF-900 furnace coupled to a LECO-CHNS-932 microanalyzer. The analyses were performed using 1mg of ground sample. The results were quoted from an average of four measurements. In all cases, the experimental error was <0.5% of the absolute value. The ash content was determined by TGA on a TA SDT 2960 analyzer under an air flow of 100 mL min^{-1}. The samples were run in heat mode at 10 ºC min^{-1} from 40 to 1000 ºC.

Raman spectroscopy: Raman spectroscopy analysis was carried out on a Micro-Raman Horiba XploRA equipment in a frequency range of 3000 to 200 cm^{-1}, using a 532 nm laser.

Thermogravimetric análisis (TG): Thermogravimetric measurements were performed using a TGA Q500 (TA Instruments) analyzer. 5 mg samples were subjected to a 10 °C min^{-1} heating ramp from room temperature to 800 °C under a 100 mL min^{-1} nitrogen flow.

X-ray diffraction (XRD): All materials were analyzed by X-ray diffraction (XRD) on a Bruker D8Advance ECO connected to a 40 kV and 25 mA power supply. Infrared spectra were acquired using a Ge crystal on an ATR Nicolet iS5 (Thermo Scientific). The crystallite height perpendicular to the graphene layers (Lc) was calculated from the (002) diffraction peak using the Scherrer equation. The corresponding number of coherently stacked graphitic

layers (N) was estimated from the equation $N = (Lc/d_{002}) + 1$, where d_{002} corresponds to the interlayer spacing calculated from the position of the (002) reflection. This parameter was used to assess the degree of graphitic stacking in the carbon precursors.

Scanning electron microscopy (SEM): The morphology of graphene oxide materials was analyzed by scanning electron microscopy (SEM) on a JEOL JCM 6000 operating at 15 kV. Sonicated ethanol suspensions of the samples were deposited on standard carbon copper grids by drop casting and observed by transmission electron microscopy. A symmetrical condenser objective lens type S-TWIN (with spherical aberration (cs=1.25 mm)) was also used.

Transmission electron microscopy (TEM). These analyses were conducted on a FEI-TITAN equipment, FEI Technology de México S.A., Monterrey, Mexico including a 200–300 kV field emission gun microscope, with a symmetrical condenser objective lens type S-TWIN (with a spherical aberration (Cs = 1.25 mm))) was also used.

S2.- Analysis of reproducibility

The data distribution was analysed by organising the measurements according to two variables: the angular velocity used during the material exfoliation process and the intensity recorded using the X-ray diffraction (XRD) equipment. The data were collected and organised in a spreadsheet using OriginLab Origin software, version 9.0. This software was then used to generate a boxplot, which made it possible to visualise the distribution of intensity values corresponding to each exfoliation speed.

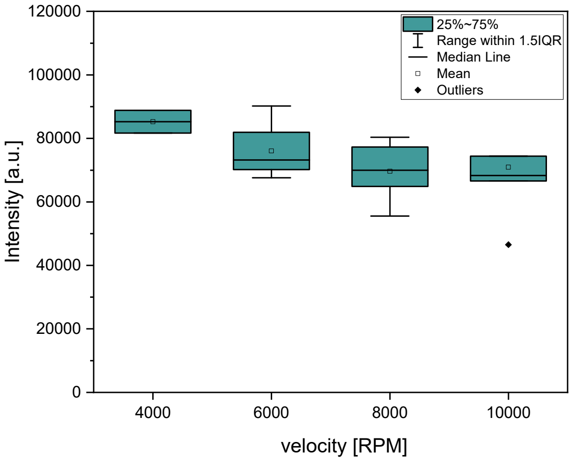

Figure S1. Boxplot representation of the X-ray intensity measured for samples obtained at different centrifugation speeds (4000, 6000, 8000 and 10,000 rpm). The boxes represent the interquartile range (25-75%), the horizontal line inside each box corresponds to the median, the square symbol indicates the mean value, whiskers extend to values within 1.5 times the interquartile range (IQR), and diamonds represent outliers.

S3.- FTIR spectra of the untreated and LPE-treated graphitic materials

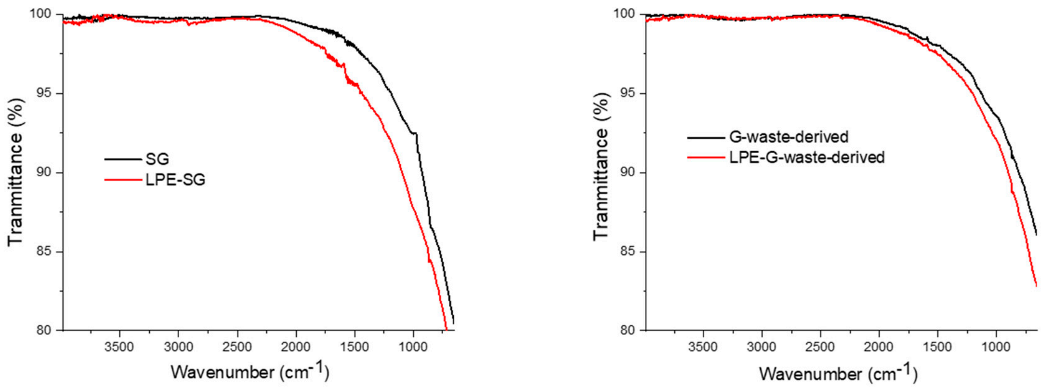

Figure S2. FTIR spectra of the untreated and LPE-treated graphitic materials.

No significant changes were observed in the characteristic bands associated with oxygen-containing functional groups after the aqueous LPE treatment, indicating that the process primarily induces structural modifications while largely preserving the original surface chemistry.
